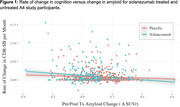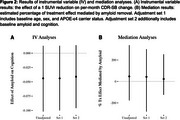# Reanalysis of the A4 Study to Formally Evaluate Amyloid Removal as a Surrogate for Cognitive Decline

**DOI:** 10.1002/alz70859_106785

**Published:** 2025-12-26

**Authors:** Sarah F Ackley, Michael Flanders, Ruijia Chen, Jingxuan Wang, Sachin J Shah

**Affiliations:** ^1^ Brown University, Providence, RI USA; ^2^ Boston University, Boston, MA USA; ^3^ University of California, San Francisco, San Francisco, CA USA; ^4^ Massachusetts General Hospital, Boston, MA USA; ^5^ Harvard Medical School, Boston, MA USA

## Abstract

**Background:**

Amyloid removal has been used as surrogate outcomes in clinical trials of Alzheimer’s disease (AD) drugs, leading to approvals of aducanumab and lecanemab under the Accelerated Approval Program. While well‐established epidemiologic and econometric methods exist to formally evaluate amyloid’s validity as a surrogate outcome, their application has been hindered by restricted access to individual‐level data from anti‐amyloid drug trials. The newly available individual‐level data from the A4 trial of solanezumab offers a unique opportunity to demonstrate how these untapped approaches can improve our understanding of the impact of amyloid removal on cognitive decline.

**Method:**

We used data on 815 A4 study participants (Alzheimer’s Clinical Trial Consortium A4/LEARN) with complete follow‐up measures for florbetapir PET imaging and Clinical Dementia Rating Scale‐Sum of Boxes (CDR‐SB) score. We estimated cognitive change using the trajectory of the CDR‐SB score over 4.5 years. Instrumental‐variable (IV) methods were used to evaluate the causal effect of amyloid reduction on cognitive changes using randomization as an instrument for amyloid reduction. Causal mediation analysis was conducted to estimate the extent to which changes in amyloid mediated the cognitive effects of solanezumab. Analyses were adjusted for sex, APOE‐ε4 carrier status, and baseline age, cognition, and amyloid.

**Result:**

Average between‐group differences in amyloid change and cognitive trajectory were ‐0.05 (SD 0.16) SUVr and 0.002 (SD 0.028) CDR‐SB points per month, respectively. Non‐linear effects of amyloid on cognition were not supported (figure 1). The IV‐estimated effect of a 1 SUVr reduction on monthly CDR‐SB change was ‐0.041, 95% CI: (‐0.096, 0.014) (figure 2). Mediation analysis suggests that amyloid change mediates 23% of solanezumab’s effect on cognitive change, 95% CI: (‐125%, 253%) (figure 2).

**Conclusion:**

Using epidemiologic and econometric methods to analyze individual‐level data from trials of amyloid‐targeting drugs will improve our understanding of amyloid as a surrogate outcome for cognition. In this instance, results are imprecise because solanezumab did not effectively remove amyloid. However, reproducing these analyses using individual‐level data from effective anti‐amyloid trials has the potential to shape treatment strategies and inform use of surrogate outcomes in future approval processes.